# Etiology, pathology, and treatment of osteonecrosis of the femoral head in adolescents: A comprehensive review

**DOI:** 10.1097/MD.0000000000039102

**Published:** 2024-07-26

**Authors:** Yuhan Lou, Jiawen Wu, Ying Zhong, Peijian Tong, Wenxi Du

**Affiliations:** a Jinhua Hospital of Traditional Chinese Medicine, Jinhua, China; b The First Affiliated Hospital of Zhejiang Chinese Medical University, Hangzhou, Zhejiang, China.

**Keywords:** adolescents, autologous stem cell transplantation, core decompression, femoral head necrosis, research progress

## Abstract

Femoral head necrosis is a common refractory disease in orthopedics, and shows a trend of getting younger. The occurrence of femoral head necrosis in adolescents is related to the use of glucocorticoids, autoimmune diseases, trauma, and other factors. Because adolescent patients are in the period of physical development, high activity requirements, and have fertility needs in the future, treatment is relatively difficult. Early artificial joint replacement may have problems such as wear and loosening, so total hip replacement is not the preferred treatment for adolescent patients with femoral head necrosis. This article will elaborate the research progress of femoral head necrosis in adolescents from 3 aspects, and summarize the benefits and side effects of core decompression combined with autologous stem cell transplantation in the treatment of early femoral head necrosis, so as to provide clinical ideas for the treatment of femoral head necrosis in adolescents.

## 1. Introduction

Osteonecrosis of the femoral head (ONFH), also known as avascular necrosis of the femoral head, is caused by hormones, alcohol, trauma, autoimmune diseases, etc, resulting in insufficient blood supply to the femoral head, causing bone tissue necrosis, repair disorders, structural changes or even collapse of the affected area. The main clinical manifestations are hip pain and dysfunction.^[1]^ According to epidemiological studies, ONFH also known as avascular necrosis of the femoral head, is caused by hormones, alcohol, trauma, autoimmune diseases, etc, resulting in insufficient blood supply to the femoral head, causing bone tissue necrosis, repair disorders, structural changes or even collapse of the affected area. The main clinical manifestations are hip pain and dysfunction.^[2]^

According to epidemiological studies, the annual incidence of ONFH in different countries has gradually increased.^[3]^ ONFH occurs in patients aged 20 to 40 years old, and the disease progresses rapidly. Most patients will have femoral head collapse. If not treated in time, it may lead to loss of hip function.^[4]^ At present, epidemiological reports on ONFH have not yet been formed, and some countries have screened their osteonecrosis populations. For example, more than 20,000 cases of ONFH are newly diagnosed in the United States each year.^[5]^ The incidence of the disease remains stable, with about 10,000 to 20,000 new cases each year.^[6]^ In addition, with the change of lifestyle, the improvement of health awareness and the improvement of diagnostic techniques, the incidence of ONFH is increasing and the incidence of ONFH is becoming younger. ONFH has become a serious worldwide health problem, which deserves our attention and attention.^[1]^ However, because teenagers are in the period of physical development, there are fertility needs in the future, and the loss of artificial joints, most patients need to undergo revision surgery twice or even multiple times, which is bound to increase the mental and economic burden of patients. Therefore, total hip arthroplasty is not the preferred treatment for adolescent ONFH patients. Therefore, how to treat adolescent ONFH is a clinical problem to be solved urgently. Therefore, this article will elaborate on femoral head necrosis in adolescents from 3 aspects: etiology, pathology, and treatment, strengthen the attention to femoral head necrosis in adolescents and provide a variety of options for its treatment. Secondly, this article will summarize the benefits and side effects of core decompression combined with autologous stem cell transplantation in the treatment of early femoral head necrosis in detail, and provide treatment ideas for early femoral head necrosis.

## 2. Etiology of femoral head necrosis in adolescents

With a large number of clinical and basic research progress, a variety of theories about the etiology of ONFH have been proposed, including abnormal lipid metabolism, intravascular coagulation, osteocyte apoptosis, osteoporosis, and genetic polymorphism changes. On the basis of etiology, blood flow damage leads to avascular necrosis of the femoral head, which causes structural damage and weight loss of the femoral head. It is considered to be the main pathogenesis.^[7]^

The etiology of ONFH can be classified according to different influencing factors, including traumatic and nontraumatic, and nontraumatic is more common in clinical practice. Most of the causes of traumatic ONFH include acetabular and femoral neck fractures, hip dislocation, and severe sprains or contusions (no fracture but joint bleeding), while nontraumatic ONFH is mainly caused by corticosteroid use, long-term excessive alcohol intake, autoimmune diseases, and coagulation disorders.^[8]^ Studies have found that the incidence of steroid-induced ONFH has increased significantly because glucocorticoids are widely used in clinical practice. According to statistics, steroid-induced ONFH is the most common type of nontraumatic ONFH.^[9]^ In addition, smoking, obesity, radiotherapy, pregnancy, and other factors are currently considered to be an important part of increasing the risk of ONFH.^[10]^

Taking corticosteroids can lead to vasoconstriction of the body, causing increased coagulation factors and fat production. At the same time, it reduces osteogenic activity and reduces bone repair and remodeling through the production of fat embolus.^[11]^ Wang et al^[12]^ listed 5 main pathogenesis theories of steroid-induced osteonecrosis of the femoral head (SONFH) in related studies: decreased osteogenic potential, lipid metabolism disorder, insufficient blood supply, cell apoptosis, and genetic polymorphisms. Related studies have shown that cortisone is an independent risk factor for osteonecrosis.^[13]^ Related studies have also shown that alcohol can weaken the activity of osteoblasts and is not conducive to bone formation.^[14]^ Therefore, hormones and alcohol have an adverse effect on bone differentiation and blood supply. However, there are also many alcoholics who are still not ill, which is a direction worthy of study. At present, there is a hypothesis that heredity is one of the pathogenesis of ONFH. In order to verify the statistical analysis of the family pedigree, the autosomal dominant inheritance of ON was found. Further studies have found that mutations in autosomal dominant genes are associated with type II collagen abnormalities, leading to the occurrence of OFNH.^[15]^ However, no available screening markers have been developed for this gene mutation.

## 3. Pathology of femoral head necrosis in adolescents

The pathological process of osteonecrosis of the femoral head mainly includes repair after osteonecrosis of the femoral head, collapse of the necrotic area after repair, and ultimately may lead to osteoarthritis, in which bone metabolism plays an important role. Bone metabolism refers to the continuous metabolism of bone cells (mainly osteocytes, osteoblasts, and osteoclasts) for bone remodeling and reconstruction, including bone formation and bone resorption. In the development of pathological process, the disease will gradually progress, and different treatment schemes should be adopted in different pathological stages. For example, in the initial stage of femoral head necrosis, oral drugs can be used for treatment, but in the collapse stage, surgical treatment should be carried out as soon as possible.

Existing studies have shown that the pathophysiological mechanism of ONFH is derived from the interaction between vascular injury, bone cell physiological changes, risk factors, and genetics.^[16]^ However, the exact pathogenesis of ONFH has not been thoroughly studied. The pathology of each theory is mainly femoral head ischemia, as well as the death and repair process of femoral head bone tissue after ischemia. The similarity is high, and the etiology is single. Therefore, with the development of scientific research, some scholars have put forward the theory of secondary collision, that is, the combination of multiple pathogenic factors. For example, Wei Biao Fang^[17]^ found that 63 of 134 patients treated with hormone therapy had ONFH, and CYP1A2G2964 A and other gene polymorphisms may be related to the genetic susceptibility of SONFH, indicating that genetic factors also play a key role in drug pathogenesis. Therefore, the understanding of the complex etiology is further enhanced, and the theory of secondary collision that advocates multifactor combined pathogenesis has also attracted everyone’s attention.

The secondary collision theory holds that the pathogenic factors of ONFH can be one or more. If the patient has a genetic susceptibility, on this basis, it is subject to risk factors ``collision,’’ which can cause ONFH. If the patient is exposed to one or more pathogenic factors for a long time, the blood is in a state of high coagulation and low fibrinolysis. If there are other dangerous causes or the cumulative tissue pressure reaches the threshold, it can cause mechanical vascular interruption, thrombotic intravascular occlusion, extravascular compression and other femoral head circulation damage, followed by ONFH.^[18]^ In recent years, the research on the genetic susceptibility of ONFH has gradually deepened, and many genetic polymorphisms have a clear effect on the pathophysiology of ONFH. Nitric oxide (NO) is a vasoactive substance that plays an important role in homeostasis, vascular system, and bone turnover. Endothelial nitric oxide synthase (eNOS) is the main subtype of NOS in normal adult bone, and is the main control gene regulating NO production.^[19]^ Koo et al^[20]^ analyzed the DNA genotype of 103 patients with nontraumatic ONFH and found that the intron 4 polymorphism of the eNOS gene was significantly associated with idiopathic ONFH. Carrying the 4a allele on it can reduce the synthesis of eNOS. At the same time, it was observed that patients with ONFH had more smokers or higher exposure to smoking than healthy controls. Glueck et al^[21]^ found that the production of NO was affected by the single nucleotide polymorphism of T-786C-eNOS, that is, the nucleotide thymine was replaced by cytosine at the 786 base pair gene site upstream of the eNOS gene, which caused the occurrence of pathological changes such as platelet aggregation, reduced angiogenesis and reduced bone formation. In addition, in the experiment of T-786C-eNOS genotype, smoking and the interaction between smoking and eNOS genotype in 95 patients with ONFH, it was also proved that smoking was an important synergistic risk of osteonecrosis associated with T-786C-eNOS polymorphism, and also provided an important basis for the feasibility of secondary collision theory. Chen et al^[22]^ conducted genetic analysis of 3 independent autosomal dominant genetic families in Taiwan and found that all affected individuals in the 3 families had glycine-serine mutations in the G-X-Y repeat sequence of the type II collagen gene (COL2A1), which made all adults carrying the COL2A1 mutant allele show groin pain and radiographic femoral head collapse, indicating that genetic susceptibility plays an important role in the pathogenesis of ONFH. Björkman et al^[23]^ studied whether Lyden’s 5th factor and prothrombin gene mutation were the pathogenic factors of nontraumatic ONFH patients. Through the investigation of 65 nontraumatic patients, it was found that the mutation rates of Lyden’s 5th factor and prothrombin 20210A gene in 35 cases of primary ONFH (without exogenous factors) were significantly higher than those in healthy control group (odds ratio 2.7, 95% confidence interval CI: 1.2–5.8) and exogenous factors group (odds ratio 10.8, 95% confidence interval CI: 1.4–84). It was observed that deep vein thrombosis was significantly increased in patients with Lyden’s 5th factor and prothrombin gene mutation, and it was analyzed that it may increase the risk of small vessel thrombosis in the femoral head due to the occurrence of related hypercoagulable state, which may lead to infarction.

On this basis, Gagala et al^[24]^ found that in addition to the above 2 genes, methylenetetrahydrofolate reductase is also associated with thrombotic diseases. In addition, the gene-tissue plasminogen activator (PLAT TPA25 I/D) associated with hypofibrinolysis has been confirmed to be a risk factor for idiopathic ONFH in the Polish population, indicating that gene polymorphisms are different among human populations. Zhu Chao et al^[25]^ searched multiple databases for SONFH-related gene polymorphisms and found that methylenetetrahydrofolate reductase C677 T gene and drug transporter (ABCB1) C3435 T gene polymorphism were significantly associated with the risk of ONFH. Patients with mutations in the above 2 genes have a greater risk of SONFH after taking glucocorticoids. Excessive use of hormones and excessive intake of alcohol can cause lipid metabolism disorders, which is an important pathogenesis leading to nontraumatic ONFH. Apolipoprotein (Apo), as an important research component of lipid metabolism, has the effect of regulating plasma lipoprotein metabolism. Wang et al^[26]^ determined that rs632153 in the ApoA1 gene was associated with an increased risk of alcohol-induced ONFH by model analysis. The rs1042034, rs676210, and rs673548 in the ApoB gene were associated with a reduced risk of alcohol-induced ONFH, and confirmed the correlation between the ApoB/ApoA1 ratio and lipid metabolism disorders. These have proved that the combination of internal and external factors or a variety of exogenous factors can easily lead to osteonecrosis. Therefore, the secondary collision theory holds that genetic susceptibility combined with multiple risk factors is synergistic in the pathogenesis of ONFH.

## 4. Iconography

In clinical practice, the early clinical symptoms and signs of avascular necrosis of the femoral head are not obvious, so it is easy to miss diagnosis and misdiagnosis. With the development of noninvasive imaging examination technology, the commonly used imaging examination techniques for the diagnosis of avascular necrosis of the femoral head are X-ray, CT, MRI, etc.^[27]^ Relevant literature points out that X-ray technology has a high diagnostic accuracy for avascular necrosis of the femoral head in the middle and late stages, but the diagnostic effect on early avascular necrosis of the femoral head is not good, and the pathological changes of the femoral head cannot be clearly displayed. The application of CT technology has high resolution and spatial resolution of bone mineral density, and can clearly observe the lesions of trabecular bone and bone capsule. However, the diagnostic accuracy of CT technology for early avascular necrosis of the femoral head with only bone marrow swelling is relatively low. During the examination of avascular necrosis of the femoral head by MRI, high resolution of soft tissue can be produced, and bone marrow edema, necrosis and granulation infiltration can be clearly displayed.^[28,29]^ According to the relevant meta-analysis, the diagnosis rate of avascular necrosis of the femoral head by MRI was higher than that by X-ray and CT.^[30]^

## 5. Treatment of femoral head necrosis in adolescents

The treatment of femoral head necrosis is mainly based on conservative treatment and surgical treatment. Conservative treatment mainly includes weight restriction, oral medication, shock wave, and hyperbaric oxygen. It has the advantages of less trauma and convenient treatment, but there are some problems such as poor treatment effect and high recurrence rate. Surgical treatment has obvious therapeutic effect, good prognosis and other clinical advantages, but the trauma is large, the patient’s acceptance is not high. The following will describe the conservative treatment and surgical treatment, respectively.

### 5.1. Expectant treatment

#### 5.1.1. Oral western medicine treatment

Changes in hemodynamics, increased blood viscosity, abnormal coagulation function, and lipid metabolism, and arteriovenous thrombosis are the main causes of ANFH formation. The drug treatment is also divided into nonsteroidal anti-inflammatory drugs, bone resorption inhibitors, cholesterol-lowering drugs, and blood coagulation prevention drugs.

##### 5.1.1.1. Nonsteroidal anti-inflammatory drugs

Nonsteroidal anti-inflammatory drugs (NSAIDs) can be called antipyretic, anti-inflammatory and analgesic drugs. NSAIDs mainly reduce the production of prostaglandin by inhibiting cyclooxygenase to achieve antipyretic, anti-inflammatory, and analgesic effects. NSAIDs can significantly reduce the pain symptoms of common orthopedic diseases (such as fractures and osteoarthritis) and are widely used in the clinical treatment of orthopedic diseases. However, NSAIDs have obvious adverse reactions, such as gastrointestinal reactions. Compared with indomethacin, the risk of selective NSAIDs (such as celecoxib, etoricoxib, etc) to the gastrointestinal tract has been significantly reduced. Even for patients with severe gastrointestinal risk, it is safe when combined with proton pump inhibitors. In addition to the above adverse reactions, NSAIDs can also increase the risk of cardiovascular events, which should be used with caution in patients with cardiovascular diseases. Wu Fengfeng et al^[31]^ have shown that nonselective NSAIDs (such as ibuprofen, aspirin, meloxicam, etc) can be used as the first choice for routine prevention of patients with high risk factors of heterotopic ossification after total hip arthroplasty. Although NSAIDs cannot reverse ANFH, they have significant effects on improving joint function and relieving pain. Relevant investigations have found that the treatment of some patients with advanced ANFH is expected to relieve symptoms and improve function. When the progress of ANFH does not reach the level that hip replacement must be performed, long-term oral medication is used. NSAIDs have a good effect on relieving pain and preventing heterotopic ossification after total hip arthroplasty in patients with ANFH.

##### 5.1.1.2. Bone resorption inhibitors

Inhibiting bone resorption drugs (such as alendronate, diethylstilbestrol, denosumab etc) mainly interfere with bone resorption by reducing the release of mature osteoclast lysosomal enzymes, thereby directly affecting the number and activity of osteoclasts, to restore the dynamic balance between osteoclasts and osteogenesis, so that the reduction of bone mineral density is reversed to achieve the purpose of delaying the progression of ANFH disease. Inhibition of bone resorption drugs in the treatment of early ANFH has a very obvious effect. At present, various studies and clinical practice show that bisphosphonates can effectively delay the progression of ANFH and have good development prospects. Han Biao et al^[32]^ found that if bisphosphonates are used as drugs to prevent revision of hip replacement in the elderly, they not only have a price advantage, but also are more easily accepted by patients. The use of drugs for a long time after hip replacement is expected to prevent the revision of joint replacement in the elderly, especially when some elderly patients are unable to tolerate the revision surgery. Anti-bone resorption drugs are widely used in clinical practice to delay the progression of early ANFH and prevent revision after joint replacement.

##### 5.1.1.3. Cholesterol-lowering drugs

Cholesterol-lowering drugs (such as simvastatin, lovastatin, ezetimibe, etc) reduce the synthesis of cholesterol in the liver and reduce the content of free cholesterol in the cells. Cholesterol-lowering drugs can effectively prevent fat embolism and microthrombus formation and delay the progression of ANFH disease. Li D et al^[33]^ found that lovastatin can promote the differentiation of bone marrow mesenchymal stem cells into osteoblasts, activate the expression of Cbfa1, and reduce the expression of adipocyte transcription factors, thereby delaying the progression of early ANFH. At the same time, recent studies have found that a variety of innovative drugs have emerged in the field of lipid-lowering drugs, such as PCSK9 monoclonal antibodies, ANGPTL antisense oligonucleotides, NPC1L1 inhibitors, etc. With the deepening of the research on cholesterol-lowering drugs, there will be more new and safe lipid-lowering drugs for the treatment of ANFH in the future.

##### 5.1.1.4. Prevention of blood clotting drugs

Anticoagulant drugs (such as warfarin, rivaroxaban, etc) improve intraosseous microcirculation by correcting blood hypercoagulability and low fibrinolysis, thereby preventing the progression of ANFH. Coagulation dysfunction can lead to intravascular coagulation and slow blood flow velocity, resulting in ischemic changes of the femoral head and eventually osteonecrosis. Dong Wei et al^[34]^ observed the X-ray results of the femoral head, the bone mineral density of the femoral head, the levels of total cholesterol and low-density lipoprotein in plasma, the levels of phosphorus, calcium and vascular endothelial growth factor in serum, and found that anticoagulation combined with lipid-lowering can improve the blood supply of the femoral head, prevent or improve embolism, and promote blood circulation and bone cell growth. Anticoagulant combined with statin lipid-lowering drugs is suitable for the treatment of ANFH caused by hormones, but it is necessary to continuously monitor coagulation function during its treatment to prevent a series of adverse reactions from excessive anticoagulant therapy.

#### 5.1.2. Cell therapy

Bone mesenchymal stem cells (BMSC) have multidirectional differentiation potential, and differentiate into osteoblasts, chondrocytes, adipocytes and so on under certain induction conditions. BMSC has the advantages of convenient sampling, strong differentiation potential, easy gene transfection, low immunogenicity and strong proliferation ability. It is the best stem cell for recent related research and clinical application. Yuan HF et al^[35]^ studied the efficacy of BMSC implantation in the treatment of femoral head necrosis, and found that BMSC implantation can improve the clinical efficacy of femoral head necrosis, delay the progression of femoral head necrosis, reduce the incidence of hip replacement and improve the Harris hip score. At the same time, BMSC combined with heterogeneous antigen-extracted cancellous bone to construct tissue-engineered bone can effectively promote angiogenesis and improve the repair effect of avascular necrosis of the femoral head.^[36]^ With the continuous research and development of cell therapy, the technology of BMSC applied to ANFH will be more perfect and mature in the future.

#### 5.1.3. Shock wave therapy

Extracorporeal shock wave therapy (ESWT) can activate the self-healing mechanism of ANFH by improving local tissue to activate stem cells, angiogenesis, cytokine release, etc., especially for early ANFH. At the same time, ESWT can be directly treated on human tissues, which can relieve local tissue inflammation and pain.^[37]^ Zhai L et al^[38]^ found that moderate-intensity ESWT can effectively promote the proliferation of stem cells and promote the differentiation of stem cells into osteoblasts by in vitro experiments on stem cells of ANFH patients. At the same time, it can reduce the probability of its differentiation into adipocytes. ESWT has the advantages of convenient operation, obvious effect and low price. It can effectively improve the quality of life of patients with ANFH and is worthy of promotion in the clinical treatment of ANFH.

#### 5.1.4. Hybaroxia

Hyperbaric oxygen can treat ANFH caused by various causes by correcting ischemia and hypoxia of tissues, improving microcirculation of local tissues, reducing blood viscosity, improving coagulation function, promoting angiogenesis, and other related mechanisms. Li Hang et al^[39]^ studied the clinical efficacy of hyperbaric oxygen therapy on medium-term steroid-induced ANFH, and found that hyperbaric oxygen therapy can improve the symptoms of hip pain in patients with medium-term ANFH and promote the repair of necrotic bone tissue. At the same time, the effect of hyperbaric oxygen combined with other therapies is very obvious. In recent years, relevant studies have shown that hyperbaric oxygen combined with electroacupuncture and hip joint training methods can alleviate the pain of patients. HBO therapy has the characteristics of simple, safe and low cost, which is helpful to delay the process of ANFH, improve the blood supply of necrotic femoral head and reduce the area of necrosis.

### 5.2. Surgical treatment

#### 5.2.1. Core decompression

Core decompression is a common surgical procedure for early ONFH. It mainly removes necrotic bone tissue by drilling in the femoral head, reduces the pressure of the medullary cavity, stimulates new bone formation, and improves the microcirculation in the necrotic area of the femoral head.^[40]^ Removal of necrotic bone tissue is a key factor in the success of core decompression.^[41]^ Core decompression has the advantages of low operation requirements, small trauma, short operation time, less bleeding, good curative effect, and does not affect the growth and development of patients.^[42]^ Clinically, it is often used to treat adolescent ONFH with stage I or II of the International Association Research Circulation osseous. A study^[43]^ found that the overall success rate of core decompression was 65 % (mean follow-up time was 54.3 months), but the therapeutic effect of surgery was also controversial. Hernandez et al^[44]^ found that core decompression has limited effect in preventing femoral head collapse and meeting the biomechanical support of femoral head. The recovery of joint function after operation is not good, and it cannot prevent the imaging progress of osteonecrosis area. Therefore, in recent years, the clinical use of core decompression alone in the treatment of ONFH is less, and more combined with bone grafting and drugs for comprehensive treatment.

#### 5.2.2. Porous tantalum rod implantation

Porous tantalum rod has certain porosity and biocompatibility, which can provide space for the attachment and regeneration of bone cells and promote new bone formation. The porous tantalum rod has high elastic modulus and compressive strength, which can meet the biomechanical support required for the femoral head. The advantages of metal rod implantation in the treatment of ONFH are: short operation time, small trauma, less bleeding, relatively simple operation, early functional exercise after operation, prevention of articular cartilage collapse, and delay of THA time. However, some studies^[45]^ have found that the incidence of femoral head collapse after porous tantalum rod implantation is 2% to 56%, and there is irreversibility after the occurrence of femoral head collapse. Although metal rod implantation has many advantages in the treatment of ONFH, its long-term efficacy has not been fully confirmed, and the risk of femoral head collapse cannot be avoided. Therefore, this procedure has been less used in clinical practice.

#### 5.2.3. Osteotomy

The basic principle of osteotomy is to change the weight-bearing area of the femoral head through surgery, thereby reducing intraosseous pressure and improving local blood supply, and providing conditions for the repair of necrotic bone tissue. At present, the common clinical osteotomy includes pelvic triple osteotomy and surgical hip dislocation. Osteotomy should be performed as early as possible in the early and middle stages of ONFH, and the necrotic lesion should not be too large, so as to reconstruct the femoral articular surface in the weight-bearing area.

##### 5.2.3.1. Triple pelvic osteotomy

Pelvic triple osteotomy is clinically used to treat developmental dysplasia of the hip, which can avoid or delay the progression of femoral head necrosis and hip arthritis. Liu Shuai et al^[46]^ performed pelvic triple osteotomy on 12 children and adolescents (19 hips) with dysplasia of the hip. The follow-up time was (34.84 ± 8.39) months. The acetabular angle, center edge angle and Reimers index at the last follow-up were improved compared with those before operation. There were 2 hips with nonunion of ischial fracture and 2 cases with unequal length of lower limbs <2 cm. By the time of the last follow-up, all patients did not progress to ONFH, and there were no complications such as neurovascular injury, infection, and broken nails. The triple pelvic osteotomy does not affect the femoral neck shaft angle and the lower limb force line, and can obtain a larger acetabular rotation range and an ideal acetabular radian, without increasing the pressure in the joint cavity, which is helpful for the recovery of joint mobility and reducing the occurrence of claudication. However, postoperative complications such as nonunion of fracture at the osteotomy site, unequal length of both lower limbs, and bone hyperplasia are prone to occur. Therefore, it is mostly used in patients with large growth and development space.

##### 5.2.3.2. Surgical hip dislocation (SHD)

SHD ensures the blood supply of the femoral head by exposing the femoral head without damaging the medial femoral circumflex artery, which is conducive to intraoperative operation and less complications of blood supply injury of the femoral head. Compared with traditional surgical methods, SHD is more safe in the treatment of hip joint injury. In recent years, the scope of application of SHD has gradually expanded, and it is often combined with bone grafting and bone flap transplantation in the treatment of ONFH. Li^[47]^ used SHD combined with bone grafting and iliac bone flap implantation to treat adolescent ONFH. The results showed that the Harris score of hip joint, the degree of femoral head collapse and the area of femoral head necrosis were significantly different from those before operation, indicating that the effect of this therapy was good. SHD has high versatility and safety for complex internal and external hip lesions, and has the advantages of sufficient intraoperative exposure, short operation time, low postoperative osteonecrosis rate, and obvious pain improvement. In addition, there is no report on the effect of SHD on the growth and development of adolescent patients. For patients with advanced adolescent ONFH, SHD-based combined with other surgeries is the preferred therapy.

#### 5.2.4. Bone grafting

Bone transplantation was used to treat ONFH, and the osteonecrosis area was first decompressed. Then the bone graft was implanted into the necrotic femoral head to reduce the high pressure in the femoral head and promote the revascularization of the graft. Then the defect was filled with artificial bone or fresh cancellous bone to induce bone cell regeneration while supporting the cartilage surface. At present, the commonly used bone grafting includes non-vascularized bone grafting and vascularized bone grafting. Bone transplantation is often combined with other surgical methods to treat ONFH. Postoperative patients need to carry out restrictive weight-bearing to promote the healing of femoral head necrosis tissue, and avoid the collapse of cartilage surface caused by weight-bearing to accelerate the process of osteonecrosis.

##### 5.2.4.1. Nonvascularized bone graft

Zuo et al^[48]^ found that the treatment of early and middle stage ONFH with SHD, sequestrum removal and impaction bone grafting can achieve satisfactory results, and this therapy has a higher success rate in young patients. Non-vascularized bone grafting requires complete removal of dead bone, but a large amount of removal of dead bone in the femoral head can reduce the biomechanical stability of the femoral head. In addition, non-vascularized bone graft has certain requirements for the blood supply of the femoral head on the affected side. At present, there are few clinical reports on non-vascularized bone grafting and the general follow-up time is short, so the medium and long-term efficacy of this therapy remains to be seen.

##### 5.2.4.2. Vascularized bone graft

Vascularized bone graft mainly includes vascularized fibula transplantation, vascularized iliac bone transplantation, and quadratus femoris bone flap transplantation. This method retains blood vessels, and the graft itself has a certain osteogenic ability, so it can promote necrotic bone healing. Fontecha et al^[49]^ found that the transplanted bone of adolescent ONFH patients survived after vascularized bone grafting, the Harris score of hip joint was higher than that before operation, and no femoral head deformation was found in the imaging examination at the last follow-up. Gorski et al^[50]^ found that after vascularized bone grafting, compared with patients with stage IV (Ficat and Arlet stage) ONFH, patients with stage II and stage III ONF had a higher bone graft survival rate. Studies^[51]^ have shown that vascularized bone graft can improve the early symptoms of ONFH, prevent the collapse of the femoral head, and the long-term effect is good.

#### 5.2.5. Bone-sparing hip replacement

For adolescent patients with ONFH, THA is not the first choice for treatment due to the long-term adverse factors such as artificial joint wear and loosening. In addition, the traditional THA needs to cut off the femoral head and femoral neck, and reaming the proximal femur, which will cause a large amount of bone loss. However, with the development of artificial prosthesis technology, the survival life of the prosthesis is relatively prolonged, and the age of the people who choose joint replacement is relatively young, and these people have higher requirements for the recovery of postoperative joint motor function. Therefore, hip replacement with bone mass preservation has become a new research hotspot.

##### 5.2.5.1. Hip resurfacing

Non-cemented acetabulum or metal-on-metal joint surface is often used in hip surface replacement. The difficulty of this operation is lower than that of THA. Due to the preservation of proximal femoral bone, higher joint mobility, and lower dislocation rate can be obtained after operation, and good long-term results can be achieved in young patients.^[52]^ Mont et al^[53]^ conducted a related study on ONFH patients who received hip surface replacement, and found that the number of people who could participate in physical activity after operation was higher than that before operation, and the amount of exercise was higher than that before operation. It was also found that the gait parameters of patients receiving hip surface replacement were better than those receiving THA. However, the metal debris produced by the wear of the hip surface replacement prosthesis has certain toxicity, and the potential long-term effects of metal ions are not fully understood. Clough et al^[54]^ found that high concentration of cobalt chromium metal is easy to cause pseudotumor syndrome around the joint prosthesis, and such metal ions can penetrate the placental barrier; therefore, metal-on-metal hip replacement is not recommended for adolescent women. Amstutz et al^[55]^ conducted a related study on 43 patients with ONFH who underwent revision surgery after hip surface replacement (with an average follow-up time of 3 years). The results showed that the hip pain and function scores after revision surgery were comparable to those after hip surface replacement, but the joint activity scores and psychological component scores after revision surgery were lower than those after hip surface replacement. The 13-year survival rate of prosthesis evaluated by Kaplan–Meier survival curve was 85.3%. The operation of hip surface replacement is difficult, the scope of application is limited, and the potential long-term effects of metal ions are not clear. Therefore, this method is rarely used in clinical practice. However, as the primary alternative to THA, hip resurfacing is very suitable for adolescent male ONFH patients with high activity demand.

##### 5.2.5.2. Short-stem hip prosthesis replacement

With the advancement of prosthesis design and surgical techniques, short-stem hip prostheses designed for anatomical reconstruction have emerged in recent years. Such prostheses have the potential to reduce bone loss and stress shielding. Hochreiter et al^[56]^ conducted a related study on 46 patients with ONFH who underwent short-stem hip prosthesis replacement (average follow-up time was 24.1 months), and found that the joint angle of the postoperative patients was maintained in the normal range, and the hip Harris score was higher than that before operation. Christiansen et al^[57]^ conducted a related study on 52 young ONFH patients who underwent ultrashort stem hip prosthesis replacement (follow-up time was 2 years), and used dual-energy X-ray absorption method to determine the bone mineral density around the prosthesis, and used radiostereoscopic measurement analysis to evaluate the degree of prosthesis displacement. The results showed that the bone mineral density of the greater trochanter and the lateral part of the femur decreased, and the bone mineral density of the lesser trochanter and the medial part of the femur increased, and the prosthesis displacement was negatively correlated with bone mineral density. Some scholars^[58]^ found that short-stem hip prosthesis cannot prevent bone loss around the prosthesis, but can keep the joint stable. The short-stem hip prosthesis has good stability at 2 years after surgery, which may help reduce bone loss around the prosthesis.

#### 5.2.6. Hip arthroscopy assisted surgery

Hip arthroscopy is a new hip-preserving method for the treatment of ONFH. It has less surgical trauma and can also evaluate the articular surface, which is helpful to judge the stage of ONFH and can also deal with some intra-articular lesions. Nazal et al^[59]^ retrospectively analyzed the case data of 8 patients (11 hips) with ONFH who underwent hip arthroscopy-assisted core decompression (follow-up time was 5 years) and found that 6 hips did not undergo THA. As far as the current research is concerned, hip arthroscopic debridement of necrotic bone can reverse or delay the process of femoral head necrosis to a certain extent, which is conducive to improving the surgical efficacy. However, the learning curve of hip arthroscopic technology is long, and the requirements for surgeons are high. There are few reports on hip arthroscopic treatment of ONFH, so the long-term efficacy of this therapy needs more data and clinical practice verification.

#### 5.2.7. Subchondral plasty

Subchondroplasty is a minimally invasive surgery developed by X-ray fluoroscopy for the treatment of early osteoarthritis, subchondral bone defects, and bone marrow lesions.^[60–64]^ Due to the collapse of the femoral head necrosis, the movement of the femoral head in the acetabulum will lead to the wear of the acetabular cartilage, causing the destruction and exfoliation of the acetabular cartilage, resulting in hip osteoarthritis. In addition, femoral head necrosis can cause local soft tissue edema and inflammation. Through the study of nontraumatic osteonecrosis of the femoral head, late and rapid destructive hip early osteoarthritis, and transient hip osteoporosis, it was found that the bone marrow changes in MR were histologically similar to edema, trabecular necrosis, and excessive stress in the subchondral bone.^[65–69]^ Therefore, it is of great significance to treat early cartilage injury with less invasiveness.^[70]^ Subchondral bone plasty supports bone repair by using orthopedic organisms that mimic the strength of subchondral bone.^[71]^ Through the experience of orthopedic treatment of tumors and fractures, it has been found that it is advisable to use synthetic bone grafts instead of autologous bone grafts.^[72–76]^ Samo K Fokter et al followed up patients with early osteoarthritis treated with tissue integration of calcium phosphate compounds after subchondral plasty. The results showed that subchondral plasty had the effect of relieving clinical symptoms, but failed to delay the natural progression of early osteoarthritis. Therefore, more patients need to be included in clinical practice for further prospective randomized studies to confirm.^[77]^

#### 5.2.8. Surgery combined with other therapies

In recent years, many new therapies for ONFH have emerged, mostly combined therapies. Wang et al^[78]^ used core decompression combined with bone morphogenetic protein bone grafting to treat ARCO stage IIIA ONFH, and found that this method can alleviate the clinical symptoms of patients and delay the imaging progress of osteonecrosis area to a certain extent. Ji et al^[79]^ used core decompression combined with artificial bone implantation and extracorporeal shock wave to treat early ONFH, and found that the efficacy of this method was better than that of simple core decompression. Bone morphogenetic protein, artificial bone and stem cell transplantation are effective in the treatment of early and middle ONFH, and can be combined with a variety of surgical treatment of ONFH, which is of great significance to delay the disease progression of ARCO stage I and II adolescent ONFH patients.

## 6. Early ONFH and core decompression combined with autologous stem cell transplantation for treatment

In the progression of ONFH, osteocyte death and trabecular bone loss are more serious, the supporting force of the weight-bearing area of the femoral head is weakened, the possibility of collapse is increased, and the therapeutic effect is significantly reduced. Therefore, early treatment of ONFH to prevent the collapse of the femoral head has important significance in protecting joint function and delaying or avoiding hip replacement. Therefore, once ONFH is diagnosed, it should be staged and classified as early as possible to formulate treatment plans, judge prognosis and evaluate efficacy. At present, the ONFH staging classification methods used in clinical practice include Ficat-Arlet, Steinberg, and ARCO staging system.^[80–82]^ The ARCO staging system revised in 2019 consists of 4 stages. On the basis of deleting stage 0, according to the severity of femoral head collapse (≤2 mm and >2 mm, respectively), the original 3 subtypes of stage 3 (collapse stage) were simplified into 2 subtypes, namely early type and progressive type. Secondly, the original quantitative index of necrotic area was deleted, that is, ONFH was no longer staged according to the size, location and length of necrotic area.^[83,84]^

Due to the development of surgery and imaging technology, ARCO developed a new classification of early (pre-collapse) ONFH in 2022.^[85]^ The necrotic lesions were divided into 3 types: type 1 was a small lesion, and the lateral necrotic edge was located inside the vertex of the femoral head; type 2 was a medium-sized lesion, and the lateral necrosis edge was located between the apex of the femoral head and the lateral edge of the acetabulum. And type 3 is a large lesion, extending to the outer edge of the acetabulum. Through a large number of clinical observations, it is found that the reliability and effectiveness of ARCO classification in 2022 are more clear and have a certain prediction of femoral head collapse. Many clinical staging methods of osteonecrosis are based on the progress of medical research at that time, and have their advantages and limitations. However, there is still no consensus on staging. Clinically, ONFH that can be treated by conservative therapy or hip-preserving surgery instead of total hip replacement, such as ARCO stage I–III, is called early ONFH.

At present, there is no consensus on the treatment of early ONFH, which usually depends on doctors based on their knowledge, skills and experience. The early clinical treatment of ONFH is also divided into nonsurgical treatment and surgical treatment. Hip preservation surgery is a common clinical treatment for early ONFH, including osteotomy and metal rod implantation. There are problems such as limited treatment conditions, poor results, poor prognosis, and easy recurrence. Therefore, the therapeutic effect, tissue recovery, and prognosis are the goals of clinical treatment. Nasser Ghaly Yousif et al^[86]^ conducted a single-center prospective study of 1994 patients with nontraumatic early (stage I–III) ONFH. The results showed that autologous hematopoietic bone marrow and CGFs transplantation combined with core decompression was beneficial for early ONFH. Relevant studies have shown that after osteonecrosis, the number of stem cells in the femoral head decreases, the ability of cell regeneration, proliferation and differentiation decreases, and the osteogenic activity decreases.^[87]^ It is beneficial to provide a sufficient number of cells and create more reasonable and effective conditions for bone repair, which is the theoretical basis for the application of bone marrow mesenchymal stem cells in the treatment of femoral head necrosis. Bone marrow mesenchymal stem cells have multi-directional differentiation potential. Under the action of bone morphogenetic protein and various growth factors, bone marrow mesenchymal stem cells differentiate into osteoblasts, which can promote bone regeneration. Secondly, the vascular growth factor secreted by bone marrow mesenchymal stem cells acts on bone marrow endothelial progenitor cells and angiogenic cells to promote vascular growth, development, and neovascularization. Furthermore, bone marrow mesenchymal stem cells produce a recruitment effect by secreting cytokines, and recruit mesenchymal stem cells to accumulate locally in the femoral head necrosis area to promote injury repair.^[88]^ In summary, bone marrow mesenchymal stem cells induce vascular differentiation by differentiating into osteoblasts, and recruit osteogenic precursor cells to the local area through the paracrine pathway to promote necrotic tissue repair. In addition, core decompression combined with autologous stem cell transplantation has the advantages of small surgical trauma, short operation time, simple operation, less surgical complications, and does not affect the later joint replacement. It has a clear advantage in the treatment of early ONFH. However, related studies have shown that core decompression combined with autologous stem cell transplantation has problems such as femoral head collapse, secondary osteoarthritis accompanied by hip pain, leading to dysfunction, and finally joint replacement. In summary, core decompression combined with autologous stem cell transplantation has the advantages of good curative effect and tissue recovery in the treatment of early ONFH, but there are also problems such as post-recovery effect. In addition, with the development and support of implanted biomaterials, it also provides more choices for the treatment of femoral head necrosis. Through the progress of science and technology, on the basis of traditional biological materials (bioceramics, bone grafts, bioglass, and polymer materials), the research and development of emerging materials such as three-dimensional (3D) printing scaffolds, nanomaterials and gene carrier materials have brought new developments for the treatment of early ONFH.

## 7. Discuss

The specific mechanism of ONFH is not completely clear, but its pathological process of femoral head necrosis mainly includes necrosis-repair-collapse-osteoarthritis. ONFH and femoral head collapse are affected by many factors. There are many treatment methods for ONFH, such as removal of etiological treatment, application of bisphosphonates, shock wave treatment, weight reduction, etc, which can effectively delay the progress of ONFH. Clinically, ONFH can be detected early by X-ray, CT, MRI, and other examinations, so as to intervene as soon as possible and avoid serious joint destruction and dysfunction.

There are many surgical methods for ONFH, but they have their own indications. We believe that: for ARCO stage I and II adolescent ONFH patients, core decompression combined with autologous stem cell implantation can be used as the first choice, and the effect is ideal. This article systematically summarizes the etiology, pathology, and treatment of ONFH, which may provide help for clinical treatment of ONFH. Surgical treatment is of great value in the treatment of ONFH in adolescents. It can not only improve the prognosis, but also avoid secondary or even multiple THA, which helps to reduce the mental and economic burden of patients. However, no matter what surgical method is used to treat ONFH, the protective weight-bearing of postoperative patients is crucial. With the development of computer technology, the application of 3D printing and surgical robots will continue to improve the accuracy and effectiveness of surgical treatment of ONFH.

## Acknowledgments

We would like to thanks Dr D, Dr T, and Dr Z for their valuable contributions to this review.

## Author contributions

**Writing – original draft:** Yuhan Lou, Jiawen Wu.

**Writing – review & editing:** Peijian Tong, Ying Zhong, Wenxi Du.

## References

[1] Zhao D Zhang F Wang B. Guidelines for clinical diagnosis and treatment of osteonecrosis of the femoral head in adults (2019 version). J Orthop Translat. 2020;21:100–10. 32309135 10.1016/j.jot.2019.12.004 PMC7152793

[2] Boontanapibul K Steere JT Amanatullah DF Huddleston JI 3rd Maloney WJ Goodman SB. Diagnosis of osteonecrosis of the femoral head: too little, too late, and independent of etiology. J Arthroplasty. 2020;35:2342–9. 32456965 10.1016/j.arth.2020.04.092

[3] Guggenbuhl P Robin F Cadiou S Albert JD. Etiology of avascular osteonecrosis of the femoral head. Morphologie. 2021;105:80–4. 33451882 10.1016/j.morpho.2020.12.002

[4] Yoon BH Mont MA Koo KH. The 2019 revised version of association research circulation osseous staging system of osteonecrosis of the femoral head. J Arthroplasty. 2020;35:933–40. 31866252 10.1016/j.arth.2019.11.029

[5] Koo KH Mont MA Cui Q. The 2021 association research circulation osseous classification for early-stage osteonecrosis of the femoral head to computed tomography-based study. J Arthroplasty. 2022;37:1074–82. 35151809 10.1016/j.arth.2022.02.009

[6] Cao H Guan H Lai Y Qin L Wang X. Review of various treatment options and potential therapies for osteonecrosis of the femoral head. J Orthop Translat. 2015;4:57–70. 30035066 10.1016/j.jot.2015.09.005 PMC5987013

[7] Delling G. Pathohistologie der Femurkopfnekrose [Pathohistology of femoral head necrosis]. Orthopade. 2007;36:404, 406–8, 410–3. German. 17450348 10.1007/s00132-007-1080-9

[8] Mont MA Pivec R Banerjee S Issa K Elmallah RK Jones LC. High-dose corticosteroid use and risk of hip osteonecrosis: meta-analysis and systematic literature review. J Arthroplasty. 2015;30:1506–12.e5. 25900167 10.1016/j.arth.2015.03.036 PMC7127809

[9] Fukushima W Fujioka M Kubo T Tamakoshi A Nagai M Hirota Y. Nationwide epidemiologic survey of idiopathic osteonecrosis of the femoral head. Clin Orthop Relat Res. 2010;468:2715–24. 20224959 10.1007/s11999-010-1292-x PMC2939331

[10] Zhang NF Li ZR Wei HY Liu ZH Hernigou P. Steroid-induced osteonecrosis: the number of lesions is related to the dosage. J Bone Joint Surg Br. 2008;90-B:1239–43. 10.1302/0301-620X.90B9.20056 18757967

[11] Kubo T Ueshima K Saito M. Clinical and basic research on steroid-induced osteonecrosis of the femoral head in Japan. J Orthop Sci. 2016;21:407–13. 27062553 10.1016/j.jos.2016.03.008

[12] Wang A Ren M Wang J. The pathogenesis of steroid-induced osteonecrosis of the femoral head: a systematic review of the literature. Gene. 2018;671:103–9. 29859289 10.1016/j.gene.2018.05.091

[13] Simon L Bertrand L Michel H Claustre BF. Osteonecrosis, alcoholism and liver steatosis. Rev Rhum Mal Osteoartic. 1975;42:103–8. 1129580

[14] Gold EW Cangemi PJ. Incidence and pathogenesis of alcohol-induced osteonecrosis of the femoral head. Clin Orthop Relat Res. 1979:222–6. 509830

[15] Yoon BH Kim TY Shin IS Lee HY Lee YJ Koo K-H. Alcohol intake and the risk of osteonecrosis of the femoral head in Japanese populations: a dose-response meta-analysis of case–control studies. Clin Rheumatol. 2017;36:2517–24. 28685377 10.1007/s10067-017-3740-4

[16] Arlet J. Nontraumatic avascular necrosis of the femoral head. Past, present, and future. Clin Orthop Relat Res. 1992:12–21. 1555331

[17] Wei B. Association of gene polymorphism with genetic susceptibility and clinical phenotype of steroid-induced osteonecrosis of the femoral head. J Shandong Med Coll. 2011;33:321–5.

[18] An FM Wang JQ Gao HY. Impact of IL1R1 and IL1R2 gene polymorphisms on risk of osteonecrosis of the femoral head from a case–control study. Mol Genet Genomic Med. 2019;7:e00557. 30623603 10.1002/mgg3.557 PMC6418375

[19] Lin RLC Sung PH Wu CT. Decreased ankyrin expression is associated with repressed eNOS signaling, cell proliferation, and osteogenic differentiation in osteonecrosis of the femoral head. J Bone Joint Surg Am. 2022;104(Suppl 2):2–12. 35389901 10.2106/JBJS.20.00465

[20] Koo KH Lee JS Lee YJ Kim K-J Yoo JJ Kim HJ. Endothelial nitric oxide synthase gene polymorphisms in patients with nontraumatic femoral head osteonecrosis. J Orthop Res. 2006;24:1722–8. 16779830 10.1002/jor.20164

[21] Glueck CJ Freiberg RA Oghene J Fontaine RN Wang P. Association between the T-786C eNOS polymorphism and idiopathic osteonecrosis of the head of the femur. J Bone Joint Surg Am. 2007;89:2460–8. 17974890 10.2106/JBJS.F.01421

[22] Chen WM Liu YF Tsai SF. Tracing the genetic origins of osteonecrosis of the femoral head. Semin Arthroplasty. 2007;18:175–9.

[23] Björkman A Svensson PJ Hillarp A Burtscher IM Rünow A Benoni G. Factor V leiden and prothrombin gene mutation: risk factors for osteonecrosis of the femoral head in adults. Clin Orthop Relat Res. 2004:168–72. 15292803

[24] Gagala J Buraczynska M Mazurkiewicz T Ksiazek A. Prevalence of genetic risk factors related with thrombophilia and hypofibrinolysis in patients with osteonecrosis of the femoral head in Poland. BMC Musculoskelet Disord. 2013;14:264. 24025446 10.1186/1471-2474-14-264 PMC3847630

[25] Zhu C Gong C Jiang L. Meta-analysis of the correlation between gene polymorphism and glucocorticoid-induced femoral head necrosis. China Mod Appl Pharm. 2019;36:2436–44.

[26] Wang Y Cao YJ Li YZ. Genetic association of the ApoB and ApoA1 gene polymorphisms with the risk for alcohol-induced osteonecrosis of femoral head. Int J Clin Exp Pathol. 2015;8:11332–9. 26617857 PMC4637673

[27] Cilla M Checa S Preininger B. Femoral head necrosis: a finite element analysis of common and novel surgical techniques. Clin Biomech (Bristol, Avon). 2017;48:49–56. 28728078 10.1016/j.clinbiomech.2017.07.005

[28] Liu Y Liang H Zhou X. Micro-computed tomography analysis of femoral head necrosis after long-term internal fixation for femoral neck fracture. Orthop Surg. 2022;14:1186–92. 35587534 10.1111/os.13318 PMC9163795

[29] Yoon YC Oh CW Kim JW Heo J Song HK. Safety of surgical hip dislocation in femoral head fracture and dislocation (FHFD) and avascular necrosis risk factor analysis of FHFD: midterm results confirmed by SPECT/CT and MRI. J Orthop Surg Res. 2022;17:278. 35578301 10.1186/s13018-022-03160-y PMC9109337

[30] Li YQ Li M Guo YM. Traction does not decrease failure of reduction and femoral head avascular necrosis in patients aged 6-24 months with developmental dysplasia of the hip treated by closed reduction: a review of 385 patients and meta-analysis. J Pediatr Orthop B. 2019;28:436–41. 30585878 10.1097/BPB.0000000000000586

[31] Wu F Gao H Huang S. Non-steroidal anti-inflammatory drugs combined with radiotherapy to prevent heterotopic ossification after total hip arthroplasty. China Orthop. 2018;31:538–42. 10.3969/j.issn.1003-0034.2018.06.011 29945410

[32] Han B Ma X Ma J. Meta-analysis of the relationship between the use of bisphosphonates and the incidence of revision arthroplasty. Chin J Bone Joint Surg. 2017;10:289–93.

[33] Li D Yang Z Wei Z Kang P. Efficacy of bisphosphonates in the treatment of femoral head osteonecrosis: a PRISMA-compliant meta-analysis of animal studies and clinical trials. Sci Rep. 2018;8:1450. 29362430 10.1038/s41598-018-19884-z PMC5780480

[34] Dong W Wang J Jia G. Study on the efficacy and mechanism of anticoagulation combined with statin lipid-lowering drugs in the treatment of steroid-induced femoral head necrosis in rat model. J Southeast Univ (Med Ed). 2019;38:131–5.

[35] Yuan HF Zhang J Guo CA Yan ZQ. Clinical outcomes of osteonecrosis of the femoral head after autologous bone marrow stem cell implantation: a meta-analysis of seven case–control studies. Clinics (Sao Paulo). 2016;71:110–3. 26934241 10.6061/clinics/2016(02)10 PMC4760366

[36] Zhang F Peng WX Wang L. Role of FGF-2 transfected bone marrow mesenchymal stem cells in engineered bone tissue for repair of avascular necrosis of femoral head in rabbits. Cell Physiol Biochem. 2018;48:773–84. 30025396 10.1159/000491906

[37] Zhai L Ma XL Jiang C Zhang B Liu ST Xing GY. Human autologous mesenchymal stem cells with extracorporeal shock wave therapy for nonunion of long bones. Indian J Orthop. 2016;50:543–50. 27746499 10.4103/0019-5413.189602 PMC5017178

[38] Zhai L Sun N Zhang B. Effects of focused extracorporeal shock waves on bone marrow mesenchymal stem cells in patients with avascular necrosis of the femoral head. Ultrasound Med Biol. 2016;42:753–62. 26674675 10.1016/j.ultrasmedbio.2015.10.021

[39] Li H Bai X Zhang Y. Clinical effect of hyperbaric oxygen therapy on steroid-induced femoral head necrosis in the middle stage. Chin J Naut Med Hyperb Med. 2018;25:94–98 + 102.

[40] Liu L Gao F Sun W. Investigating clinical failure of core decompression with autologous bone marrow mononuclear cells grafting for the treatment of non-traumatic osteonecrosis of the femoral head. Int Orthop. 2018;42:1575–83. 29654394 10.1007/s00264-018-3918-7

[41] Mont MA Carbone JJ Fairbank AC. Core decompression versus nonoperative management for osteonecrosis of the hip. Clin Orthop Relat Res. 1996;324:169–78. 10.1097/00003086-199603000-00020 8595753

[42] Herrera-Soto JA Price CT. Core decompression for juvenile osteonecrosis. Orthop Clin North Am. 2011;42:429–36, ix. 21742155 10.1016/j.ocl.2011.04.004

[43] Hua KC Yang XG Feng JT. The efficacy and safety of core decompression for the treatment of femoral head necrosis: a systematic review and meta-analysis. J Orthop Surg Res. 2019;14:306. 31511030 10.1186/s13018-019-1359-7 PMC6737645

[44] Hernandez A Nuñez JH Sallent A Gargallo-Margarit A Gallardo-Calero I Barro V. Core decompression combined with implantation of autologous bone marrow concentrate with tricalcium phosphate does not prevent radiographic progression in early stage osteonecrosis of the hip. Clin Orthop Surg. 2020;12:151–7. 32489535 10.4055/cios19033 PMC7237257

[45] Huang W Gong X Sandiford S. Outcome after a new porous tantalum rod implantation for treatment of early-stage femoral head osteonecrosis. Ann Transl Med. 2019;7:441. 31700877 10.21037/atm.2019.08.86 PMC6803177

[46] Liu S Zhang M Li T. Three-dimensional CT-assisted pelvic triple osteotomy for the treatment of developmental dysplasia of the hip in older children. Chin J Orthop. 2020;40:1165–74.

[47] Li X. Clinical characteristics of femoral head necrosis after trauma in adolescents and the significance of hip-preserving surgery to improve the therapeutic effect. World Compos Med. 2021;7:122–4.

[48] Zuo W Sun W Zhao D Gao F Su Y Li Z. Investigating clinical failure of bone grafting through a window at the femoral head neck junction surgery for the treatment of osteonecrosis of the femoral head. PLoS One. 2016;11:e0156903. 27285821 10.1371/journal.pone.0156903 PMC4902236

[49] Fontecha CG Roca I Barber I. Femoral head bone viability after free vascularized fibular grafting for osteonecrosis: SPECT/CT study. Microsurgery. 2016;36:573–7. 26214835 10.1002/micr.22452

[50] Gorski SM Dong C Krieg AH Haug M. Vascularized bone graft reconstruction following bone tumor resection at a multidisciplinary sarcoma center: outcome analysis. Anticancer Res. 2021;41:5015–23. 34593450 10.21873/anticanres.15316

[51] Lei P Du W Liu H. Free vascularized iliac bone flap based on deep circumflex iliac vessels graft for the treatment of osteonecrosis of femoral head. J Orthop Surg Res. 2019;14:397. 31779640 10.1186/s13018-019-1440-2 PMC6883673

[52] Scholes CJ Ebrahimi M Farah SB. The outcome and survival of metal-on-metal hip resurfacing in patients aged less than 50 years: a prospective observational cohort study with minimum ten-year follow-up. Bone Joint J. 2019;101-B:113–20. 30601056 10.1302/0301-620X.101B1.BJJ-2018-0702.R1

[53] Mont MA Seyler TM Ragland PS Starr R Erhart J Bhave A. Gait analysis of patients with resurfacing hip arthroplasty compared with hip osteoarthritis and standard total hip arthroplasty. J Arthroplasty. 2007;22:100–8. 10.1016/j.arth.2006.03.010 17197316

[54] Clough EJ Clough TM. Metal on metal hip resurfacing arthroplasty: where are we now? J Orthop. 2020;23:123–7. 33488008 10.1016/j.jor.2020.12.036 PMC7809170

[55] Amstutz HC Le Duff M. What are the results of revised hip resurfacing arthroplasties? Bone Joint J. 2020;102-B:1289–96. 32993340 10.1302/0301-620X.102B10.BJJ-2020-0147.R2

[56] Hochreiter J Mattiassich G Ortmaier R Steinmair M Anderl C. Femoral bone remodeling after short-stem total hip arthroplasty: a prospective densitometric study. Int Orthop. 2020;44:753–9. 31965311 10.1007/s00264-020-04486-0

[57] Christiansen JD Ejaz A Nielsen PT Laursen M. An ultra-short femoral neck-preserving hip prosthesis: a 2-year follow-up study with radiostereometric analysis and dual X-ray absorptiometry in a stepwise introduction. J Bone Joint Surg Am. 2020;102:128–36. 31596796 10.2106/JBJS.19.00104

[58] Nyström A Kiritopoulos D Mallmin H Lazarinis S. Continuous periprosthetic bone loss but preserved stability for a collum femoris-preserving stem: follow-up of a prospective cohort study of 21 patients with dualenergy X-ray absorptiometry and radiostereometric analysis with minimum 8 years of follow-up. Acta Orthop. 2022;93:206–11. 34984482 10.2340/17453674.2021.1080 PMC8815616

[59] Nazal MR Parsa A Gibbs JS Abraham PF Martin SD. Mid-term results of arthroscopic synovectomy for pigmented villonodular synovitis of the hip. Arthroscopy. 2020;36:1587–98. 32061973 10.1016/j.arthro.2020.01.059

[60] Pasqualotto S Sgroi AV Causero A Di Benedetto P Zorzi C. Subchondroplasty in the treatment of bone marrow lesions of the knee: preliminary experience on first 15 patients. Joints. 2021;7:174–81. 34235382 10.1055/s-0041-1730984 PMC8253614

[61] Cohen SB Sharkey PF. Subchondroplasty for treating bone marrow lesions. J Knee Surg. 2016;29:555–63. 26641077 10.1055/s-0035-1568988

[62] Zeng GJ Foong WS Lie TTD. Knee subchondroplasty for management of subchondral bone cysts: a novel treatment method. Singapore Med J. 2021;62:492–6. 35001129 10.11622/smedj.2021145 PMC9251239

[63] Bonadio MB Giglio PN Helito CP Pécora JR Camanho GL Demange MK. Subchondroplasty for treating bone marrow lesions in the knee-initial experience. Rev Bras Ortop. 2017;52:325–30. 28702392 10.1016/j.rboe.2017.04.003 PMC5497019

[64] Levy AS Cousins K. The rational for and efficacy of subchondroplasty in the injured worker. J Orthop. 2020;22:48–52. 32280168 10.1016/j.jor.2020.03.047 PMC7138934

[65] Tarantino U Greggi C Cariati I. Bone marrow edema: overview of etiology and treatment strategies. J Bone Joint Surg Am. 2022;104:189–200. 34780382 10.2106/JBJS.21.00300

[66] Sugano N Kubo T Takaoka K. Diagnostic criteria for non-traumatic osteonecrosis of the femoral head. A multicentre study. J Bone Joint Surg Br. 1999;81:590–5. 10463726 10.1302/0301-620x.81b4.9393

[67] Boutry N Paul C Leroy X Fredoux D Migaud H Cotten A. Rapidly destructive osteoarthritis of the hip: MR imaging findings. AJR Am J Roentgenol. 2002;179:657–63. 12185038 10.2214/ajr.179.3.1790657

[68] Watanabe W Itoi E Yamada S. Early MRI findings of rapidly destructive coxarthrosis. Skeletal Radiol. 2002;31:35–8. 11807591 10.1007/s00256-001-0445-0

[69] Vande Berg BC Lecouvet FE Koutaissoff S Simoni P Malghem J. Bone marrow edema of the femoral head and transient osteoporosis of the hip. Eur J Radiol. 2008;67:68–77. 18468828 10.1016/j.ejrad.2008.01.061

[70] Kurtz S Ong K Lau E Mowat F Halpern M. Projections of primary and revision hip and knee arthroplasty in the United States from 2005 to 2030. J Bone Joint Surg Am. 2007;89:780–5. 17403800 10.2106/JBJS.F.00222

[71] Brimmo OA Bozynski CC Cook CR. Subchondroplasty for the treatment of post-traumatic bone marrow lesions of the medial femoral condyle in a pre-clinical canine model. J Orthop Res. 2018;36:2709–17. 29748965 10.1002/jor.24046

[72] Bajammal SS Zlowodzki M Lelwica A. The use of calcium phosphate bone cement in fracture treatment. A meta-analysis of randomized trials. J Bone Joint Surg Am. 2008;90:1186–96. 18519310 10.2106/JBJS.G.00241

[73] Kim JK Kim NK. Curettage and calcium phosphate bone cement injection for the treatment of enchondroma of the finger. Hand Surg. 2012;17:65–70. 22351535 10.1142/S0218810412500104

[74] Evaniew N Tan V Parasu N. Use of a calcium sulfate-calcium phosphate synthetic bone graft composite in the surgical management of primary bone tumors. Orthopedics. 2013;36:e216–22. 23380017 10.3928/01477447-20130122-25

[75] Winge MI Reikerås O Røkkum M. Calcium phosphate bone cement: a possible alternative to autologous bone graft. A radiological and biomechanical comparison in rat tibial bone. Arch Orthop Trauma Surg. 2011;131:1035–41. 21305309 10.1007/s00402-011-1271-z

[76] Akhavan S Martinkovich SC Kasik C DeMeo PJ. Bone marrow edema, clinical significance, and treatment options: a review. J Am Acad Orthop Surg. 2020;28:e888–99. 32701688 10.5435/JAAOS-D-20-00142

[77] Fokter SK Kuhta M Hojnik M Ledinek Z Kostanjšek R. Tissue integration of calcium phosphate compound after subchondroplasty: 4-year follow-up in a 76-year-old female patient. Bioengineering (Basel). 2023;10:208. 36829702 10.3390/bioengineering10020208 PMC9952516

[78] Wang Y Li P Huang S. Mid-term efficacy of core decompression combined with bone morphogenetic protein-containing impaction bone grafting in the treatment of ARCO stage IIIA osteonecrosis of the femoral head. Chin J Bone Joint Surg. 2022;15:24–30.

[79] Ji Song J Zhang L Xu Li. The efficacy of modified core decompression and bone grafting combined with extracorporeal shock wave in the treatment of early femoral head necrosis. Pract Med J. 2022;38:1913–8.

[80] Zhang HX Zhang XP Xiao GY. In vitro and in vivo evaluation of calcium phosphate composite scaffolds containing BMP-VEGF loaded PLGA microspheres for the treatment of avascular necrosis of the femoral head. Mater Sci Eng C Mater Biol Appl. 2016;60:298–307. 26706534 10.1016/j.msec.2015.11.055

[81] Gu Y Wang G Zhang X. Biodegradable borosilicate bioactive glass scaffolds with a trabecular microstructure for bone repair. Mater Sci Eng C Mater Biol Appl. 2014;36:294–300. 24433915 10.1016/j.msec.2013.12.023

[82] Ikeuchi K Hasegawa Y Seki T Takegami Y Amano T Ishiguro N. Epidemiology of nontraumatic osteonecrosis of the femoral head in Japan. Mod Rheumatol. 2015;25:278–81. 25036228 10.3109/14397595.2014.932038

[83] Tani T Ando W Fukushima W. Geographic distribution of the incidence of osteonecrosis of the femoral head in Japan and its relation to smoking prevalence. Mod Rheumatol. 2022;32:186–92. 33719872 10.1080/14397595.2021.1899452

[84] Sato R Ando W Fukushima W. Epidemiological study of osteonecrosis of the femoral head using the national registry of designated intractable diseases in Japan. Mod Rheumatol. 2022;32:808–14. 34910162 10.1093/mr/roab047

[85] Che Z Song Y Zhu L Liu T Li X Huang L. Emerging roles of growth factors in osteonecrosis of the femoral head. Front Genet. 2022;13:1037190. 36452155 10.3389/fgene.2022.1037190 PMC9702520

[86] Suo Yousif NG Al Kilabi AEK Hatem KK. Autologous hematopoietic bone marrow and concentrated growth factor transplantation combined with core decompression in patients with avascular necrosis of the femoral head. J Med Life. 2023;16:76–90. 36873113 10.25122/jml-2022-0342 PMC9979168

[87] Jeyaraman M Muthu S Jain R Khanna M. Autologous bone marrow derived mesenchymal stem cell therapy for osteonecrosis of femoral head: a system overview of overlapping metaanalyses. J Clin Orthop Trauma. 2020;13:134–42. 33717885 10.1016/j.jcot.2020.11.015 PMC7920111

[88] Xu Y Jiang Y Xia C Wang Y Zhao Z Li T. Stem cell therapy for osteonecrosis of femoral head: opportunities and challenges. Regen Ther. 2020;15:295–304. 33426232 10.1016/j.reth.2020.11.003 PMC7770428

